# The Role of Antennae in Removing Entomopathogenic Fungi from Cuticle of the Termite, *Coptotermes formosanus*

**DOI:** 10.1673/031.009.0601

**Published:** 2009-03-17

**Authors:** Aya Yanagawa, Fumio Yokohari, Susumu Shimizu

**Affiliations:** ^1^ Institute of Biological Control, Graduate School of Bioenvironmental Science, Kyushu University, Fukuoka 812-8581, Japan; ^2^ Research Fellowships of the Japan Society for the Promotion of Science for Young Scientists; ^3^ Division of Biology, Department of Earth Science, Faculty of Science, Fukuoka University, Fukuoka 814-0180, Japan

**Keywords:** termite, mutual grooming behavior, entomopathogenic fungi, antennae, electroantennogram response, *Beauveria brongniartii* 782, *Paecilomyces fumosoroseus* K3, *Metarhizium anisopliae* 455

## Abstract

Our previous research has shown that the termite, *Coptotermes formosanus* Shiraki (Isoptera: Rhinotermitidae), protects itself from entomopathogenic fungi by mutual grooming behavior. The termite removes and discards foreign organisms, such as fungal conidia, from the body surface of its nestmates by mutual grooming behavior. The role of the antennae in detecting the condia was examind here. Three entomopathogenic fungi were used, *Beauveria brongniartii* 782 (Saccardo) (Hypocreales), *Paecilomyces fumosoroseus* K3 (Wize) (Hyphomycetes), and *Metarhizium anisopliae* 455 Sorokin (Hyphomycetes). Termites with antennae removed conidia more efficiently than termites without antennae. There were differences between termites with and without antennae in selection of sites to be groomed on nestmates, in the length of grooming and in occurrence of grooming. Electroantennogram (EAG) responses were recorded from termite antennae and the waveforms were rather specific to the kinds of fungi used as odor sources. Termites were able to distinguish between the tested fungi in feeding tests. These results show that the antennae play important roles in the mutual grooming behavior of the termite.

## Introduction

The termite *Coptotermes formosanus* Shiraki (Isoptera: Rhinotermitidae) is one of the most destructive insects of houses and wooden structures in Japan (Vargo et al. 2003). Although chemicals have been traditionally used for termite control, biological control with entomopathogenic fungi is an important alternative to chemical control (Sun et al. 2002). As termites live in high-density populations and in high humidity habitats, fungi are expected to be useful for biological pest control against termites. However, fungal epizootics of termite populations are not well defined yet, even though most soil contains resident populations of many species of entomopathogenic fungi (Yaginuma 1990). It is, therefore, useful to study the relationship between termites and entomopathogenic fungi to develop them as sustainable biological pest control agents.

The mutual grooming behavior of workers of *C. formosanus* is very effective against fungal infection (Yanagawa and Shimizu 2005). Termite workers remove foreign organisms from the body surface of their nestmates with glossae, eat and excrete them. Thus, termites are highly resistant to entomopathogenic fungi within the colony. Various species of termites including *C. formosanus, Reticulitermes speratus* and *R. flavipes* mutually groom in a similar manner and are also highly resistant to entomopathogenic fungi (Yanagawa and Shimizu 2005; Boucias et al. 1996; Shimizu and Yamaji 2003). These facts strongly suggest that the mutual grooming is a disease-defensive social behavior in termites. Indeed, most social insects frequently engage in mutual grooming thereby cleaning the bodies of their nestmates (Hefetz et al. 2001; Hughes et al. 2002; Fussnecker et al. 2006).

As for the induction of mutual grooming, antennal contact with nestmates has been suggested to be essential (Dhanarajan 1980). Myles (2002) also suggested that antennal contact could set off social behavior in termites. However, in our preliminary experiment, termites with both antennae entirely removed, frequently performed mutual grooming. The role of antennae in induction of grooming behavior in termites therefore remains ambiguous.

To protect against fungal infection, the termites need to recognize pathogenic conidia on the body surface and then to remove them. In this context, we estimated in this study some roles of antennae related with mutual grooming. The results suggest that termite antennae play some essential roles in recognition of the conidia, in the selection of grooming sites and in the length of grooming behavior. This paper also provides the first electroantennogram (EAG) responses of termites to odors of entomopathogenic fungi.

## Materials and Methods

### Insects

Termites, *C. formosanus*, were collected in Fukuoka, Japan and maintained in plastic boxes (49 × 36 × 32 cm) in a dark chamber at 25 °C, and were fed on seasoned pinewood. Before being used in behavioral experiments, worker termites were transferred from the above boxes into Petri dishes (90 × 15 mm; 20 termites per dish), which contained a wet paper disc, and were placed in the dark chamber at 25 °C for 1 to 3 weeks. Specimens used in the electrophysiological experiment were supplied by the Faculty of Agriculture, Yamaguchi University, Japan, and had been kept in the condition described above.

### Preparation of conidial suspensions

Entomopathogenic fungi, *Beauveria brongniartii* 782 (Saccardo) (Hypocreales) and *Paecilomyces fumosoroseus* K3 (Wize) (Hyphomycetes) were maintained on L-broth agar (polypeptone, 1%; yeast extract, 0.3%; sucrose, 2.0%; NaCl, 0.5%; agar, 2.0%) at 25 °C. *Metarhizium anisopliae* 455 Sorokin (Hyphomycetes) was maintained on potato dextrose agar (potato extract, 0.4%; glucose, 2.0%; agar, 1.5%) at 25 °C. Conidia were harvested with a brush from 10- to 15-day-old cultures.

Conidia of *M. anisopliae* 455 were surface-labeled with fluorescent isothiocyanate (FITC) (Sigma Chemical, www.sigmaaldrich.com) to visualize them on the body surface of the termites. Conidia of *M. anisopliae* 455 were suspended in a 0.025% aqueous solution of Tween 20 (0.025% Tween 20 solution) and surface-labeled with a 0.01% FITC solution according to Hung and Boucias (1992). FITC-labeled conidia in a 0.025% Tween 20 solution were counted with a Thoma hemocytometer (Reichert, www.reichert.com) and adjusted to the concentration of 1.0 × 10^7^ conidia/ml (A series).

To study EAG responses to the odor of conidia of entomopathogenic fungi, conidial suspensions of *B. brongniartii* 782, *P. fumosoroseus* K3 and *M. anisopliae* 455 were prepared in 0.025% Tween 20 solutions and were adjusted to the concentration of 1.0 × 10^7^ conidia/ml as described above (B series).

### Removal of *M. anisopliae* conidia attached to the surface of termites with or without antennae

Termites with and without antennae were used. To prepare termites with no antennae, both antennae were cut off at the scape after termites had been cold anesthetized on ice for 30 minutes. Termites with no antennae were maintained for two days to allow the antennal cut ends to be healed, before they were inoculated with conidia. For inoculation, termites with and without antennae were each put into a microcentrifuge tube containing the FITC-labeled *M. anisopliae* 455 conidial suspensions (A series). The termites were submerged in the conidial suspensions with gentle swirling for 5 seconds, removed from the tube and dried on filter paper. The termites were then washed once in 0.025% Tween 20 solution to remove the nonattached conidia. Ten termites were then put together into Petri dishes (90 × 15 mm) containing a wet paper disc and reared at 25 °C. Ten termites with and without antennae were sampled at 0, 3, 6 and 24 hours after inoculation and stored at -20 °C. The stored termites were mounted in a drop of Vectashield (Vector Laboratories, www.vectorlabs.com) to stabilize the fluorescence and the number of conidia were counted on five defined sites of the termite surface (head, thorax and the 2nd, 4th and 6th abdominal segments) using an epifluoresent microscope (Carl Zeiss, www.zeiss.com) at 200 ×.

### EAG response of termite antennae

An antenna excised at scape, as described above, was fixed on a slide glass with double-sided adhesive tape under a standard dissecting microscope. Both ends of the antenna were inserted slightly into the glass electrodes, enough to make an electrical contact, and thereafter shielded with liquid paraffin in order to prevent fluid in the electrodes and lymph in the antenna from evaporating. The fluid in the electrodes was physiological saline for cockroaches (1.5 % 200 mM KC10.9 %; 200 mM CaCl_2_-2H_2_O; 0.1 % 200 mM Na_2_HPO_4_2H_2_O; 0.9 % 200 mM NaH_2_PO_4_·H_2_O) (Yamazaki and Narahashi 1959). The electrodes were made from borosilicate glass tubes (0.50 mm ID, 1.0mm OD) using a laser puller (Sutter Instruments, http://www.sutter.com).

The stimulus and control air were prepared as follows. Fresh air was taken by a diaphragm pump from the outside. The air was desiccated with silica gel and then cleaned by passing through active carbon. The flux was controlled at 1.0 liter/minute by a flowmeter en route. The cleaned air was then fed to a three-way electromagnetic valve operated by an electric pulse generator. One of the outlets was connected to a glass tube for the control stream and the other was further divided into four branches by means of glass T tubes. Each branch was connected to a small glass bottle (30 ml) after passing through a stop valve that was used to select the stimulus. Each bottle contained a different kind of odor substance, as described later. The air passing through these bottles was separately fed to glass tubes for stimulation. The nozzles (3 mm in inner diameter) of the control and stimulus tubes were arranged 2 cm apart from the specimen in a concentric circle. The stimulus interval was set as at least three minutes because it usually takes 2–3 minutes for the olfactory receptors of insects to recover their full excitability following stimulation. The duration of one stimulus puff was set at 2 seconds. The specimen was exposed to the control clean air during the interstimulus time. In order to keep the experimental environment clean the air near the sample was always ventilated.

**Table 1.**
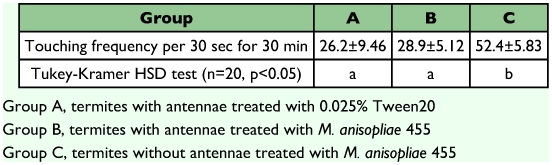
Touching frequency of the termites group (A), (B), and (C)

The contents of the four bottles were as follows. One bottle contained 1 ml of 0.025% Tween 20 solution without any suspension. Each of the others contained one of 1 ml conidial suspensions of 1.0 × 10^7^ conidia/ml of *B. brongniartii* 782, *P. fumosoroseus* K3 or *M. anisopliae* 455 in 0.025% Tween 20 solutions (B series). In the experiment to study the concentration effects of *M. anisopliae* 455, conidial suspensions of 10^3^, 10^4^, 10^5^, 10^6^ or 10^7^ conidia/ml were each put into one of the bottles.

### Statistical analysis

Conidia removal of the termite with and without antennae was analyzed by Poisson regression models using SAS (version 9.1) (Proc GENMOD, SAS Institute 1999). To evaluate statistical significance among EAG responses, the Tukey-Kramer HSD test was applied.

## Results

### Removal of *M. anlsopllae* conidia attached to the body surface of termites with or without antennae

In the control experiment, grooming activities were compared between normal termites and termites without antennae that had been infected with *M. anisopliae* 455. As shown in Table 1, antennal removal significantly enhanced the grooming activity of termites.

**Figure 1.**
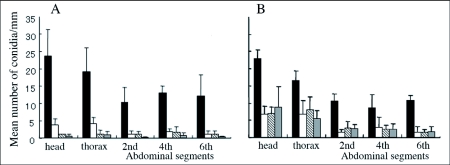
Comparison of attachment and persistence of *Metarhizium anisopliae* 455 conidia on the termite surface between termites with (A) and without (B) antennae. Black bars: termites at 0 hour post-inoculation. White bars: termites at 3 hours post-inoculation. Hatched bars: termites at 6 hours post-inoculation. Gray bars: termites at 24 hours post-inoculation.Vertical bar represents standard errors.

Termites with and without antennae were sampled 0, 3, 6 and 24 hours after inoculation (Figure 1). In termites with antennae, the number of conidia on each site of the body surface decreased drastically during the first 3 hours after inoculation and thereafter also decreased gradually (Figure 1A). Finally, the average number of conidia at all five defined sites became less than 1 /mm^2^ 24 hours after inoculation. On the other hand, in termites with no antennae, the number decreased during the first 3 hours after inoculation, but did not decrease beyond 6 hours after inoculation (Figure 1B). These figures suggest that antennae have important roles in the removal of conidia.

The difference in conidia reduction between the termites with antennae and ones without antennae were distinct, especially after the first 3 hours after inoculation (Figure 1). The data obtained at 3, 6 and 24 hours after inoculation were analyzed statistically using Poisson regression. As shown in Table 2 (a), there was no significant difference in conidia reduction between the termites with and without antennae (p = 0.0727 in-group parameter). In both termite groups, conidia were removed effectively during the first 3 hours after inoculation (Table 2 (a), p < 0.0001 in time parameter). The difference of conidia reduction between the termites with and without antennae was observed in later time intervals (Table 2 (b), p = 0.0002 in group parameter). In order to understand the detailed features of the statistical results shown in Table 2, the experimental results of termites with and without antennae were analyzed separately using Poisson regression (Tables 3 and 4). In the group of termites with antennae, the removal rate of conidia was significant regardless of the body sites in the early stage (Table 3a, p = 0.0176 in site parameter), and became non-significant in the later stages (Table 3b, p = 0.1999 in site parameter). These data suggest that termites with antennae removed conidia evenly from all sites of the body surface. Variation in removal of condia was compared between termites with and without antennae. Termites with antennae removed conidia at a similar rate during all stages (Table 3 a, b), (p < 0.0001 and p = 0.0813, respectively, in time parameters). In contrast, termites without antennae removed conidia unevenly from all sites of body surface in all stages (Table 4 (a) and (b), p < 0.0001 in site parameters). It appeared that they removed conidia only from easily accessible sites. The conidia removal in termites with no antennae was time dependent in the first 3 hours (Table 4 a), (p = 0.0008 in time parameter) but it was not thereafter (Table 4 b), (p = 0.9639 in time parameter) in contrast to that in termites with antennae. Figure 1 b also shows that the number of conidia decreased drastically in the first stage but did not thereafter. The survival time of termites after the inoculation with *M. anisopliae* 455 was compared between the termites with antennae and those without antennae. Termites without antennae all died in less than one day after inoculation, probably due to the infection of *M. anisopliae*, but most termites with antennae survived longer than 4 days (Table 5).

### EAG response of the termites

**Figure 2.**
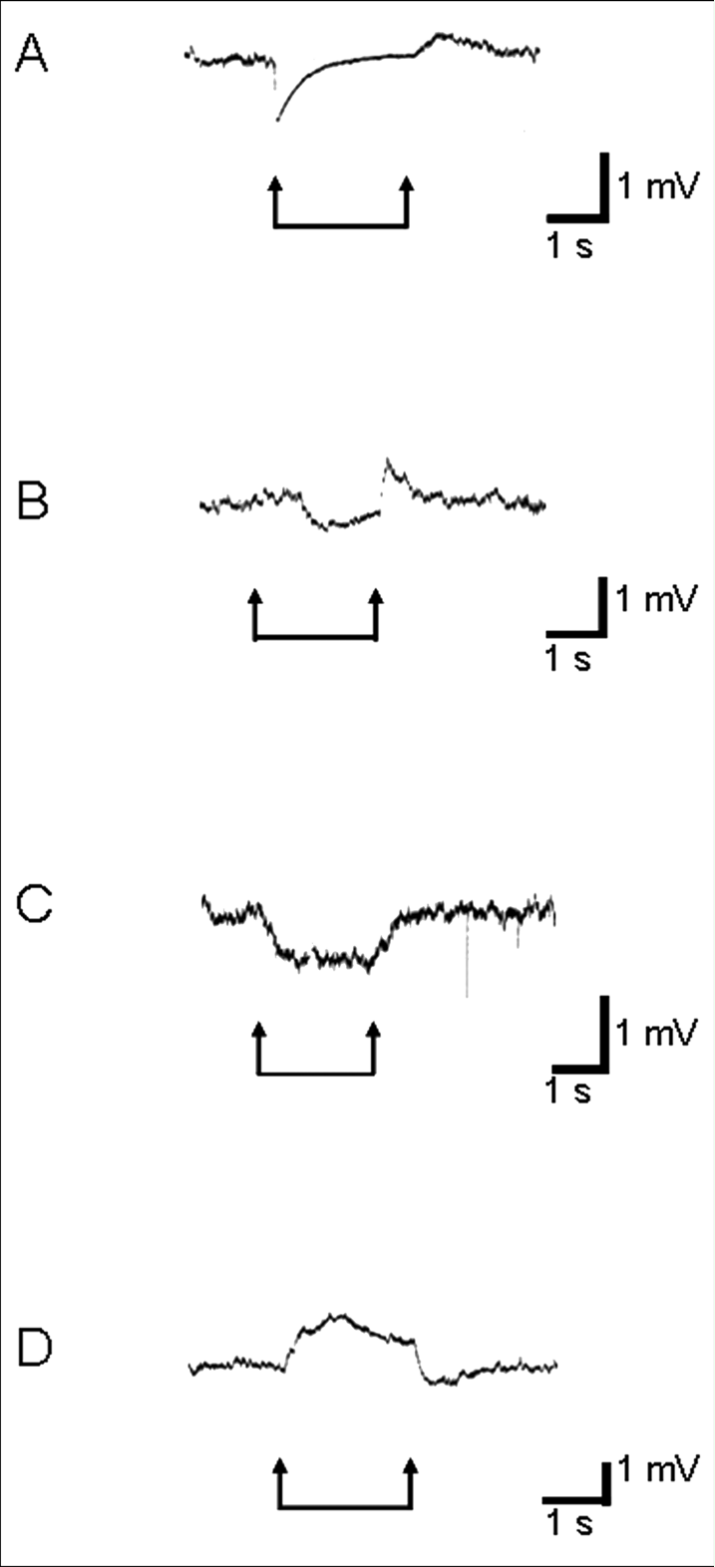
Typical EAG responses of *Coptotermes formosanus* to odor originating from suspensions of three species of entomopathogenic fungi and Tween 20 (control).EAG waveforms appear to be specific to the odor. A, EAG to Tween 20 (control); B, EAG to *Metarhizium anisopliae* 455; C, EAG to *Paecilomyces fumosoroseus* K3; D, EAG to *Beauveria brongniartii* 782. A horizontal bar with arrows under each record is the stimulation mark.

In order to examine the roles of the antennae in grooming behavior, the electrical responses of the antennae (EAGs) to odor originating from three kinds of entomopathogenic fungi were recorded (Figure 2). Twenty-five EAG responses were recorded from 5 antennae. The stimulus sources were 1 ml conidial suspensions of 1.0 × 10^7^ conidia/ml of the 3 kinds of fungi in 0.025% Tween 20 solutions. Figure 2 shows typical EAG responses to these odors. As the odor sources used in this study contained 0.025% Tween 20 solution, the response to this solution was first recorded (Figure 2A). The EAG response to a single puff (2 seconds) of 0.025% Tween 20 solution consisted of initial fast downward (negative) deflection, gradual upward (positive) deflection to the prestimulus level and further upward deflection after stimulation. The EAG response to the odor of *M. anisopliae* 455 deflected downwards after short delay followed by a sudden upward deflection at stimulus cessation (Figure 2B). The response to *P. jumosoroseus* K3 deflected gradually in downward direction in the initial phase of stimulation and maintained its potential up to stimulus cessation. This was followed by gradual recovery to the initial level after stimulus cessation (Figure 2C). The response to *B. brongniartii* 782 deflected gradually in upward direction in the initial phase and after the appearance of a blunt peak it gradually deflected downward. A sudden downward deflection then appeared at stimulus cessation (Figure 2D).

**Table 5.**
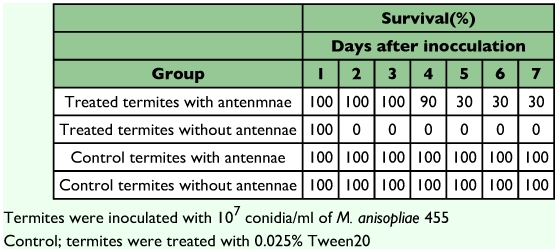
Survival of the termite with/without antennae following inoculation with *M. anisopliae* 455

**Table 2.**
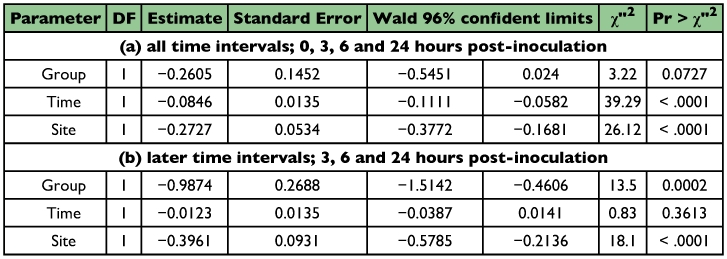
Attachment and persistence of *M. anisopliae* conidia on cuticle of the termites with/without antennae. Results of Poisson regression

**Table 3.**
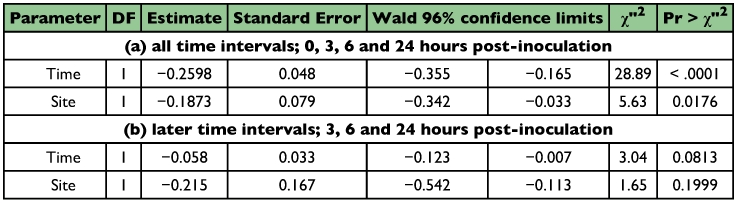
Attachment and persistence of *M. anisopliae* conidia on cuticle of the termites with antennae. Results of Poisson regression.

**Table 4.**
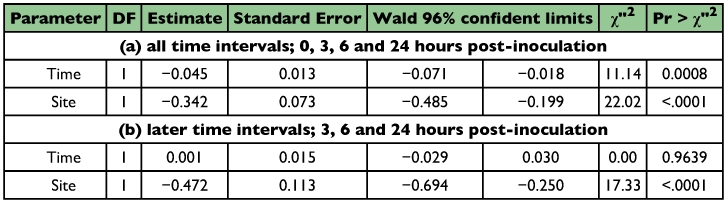
Attachment and persistence of *M. anisopliae* conidia on cuticle of the termites without antennae. Results of Poisson regression

**Figure 3.**
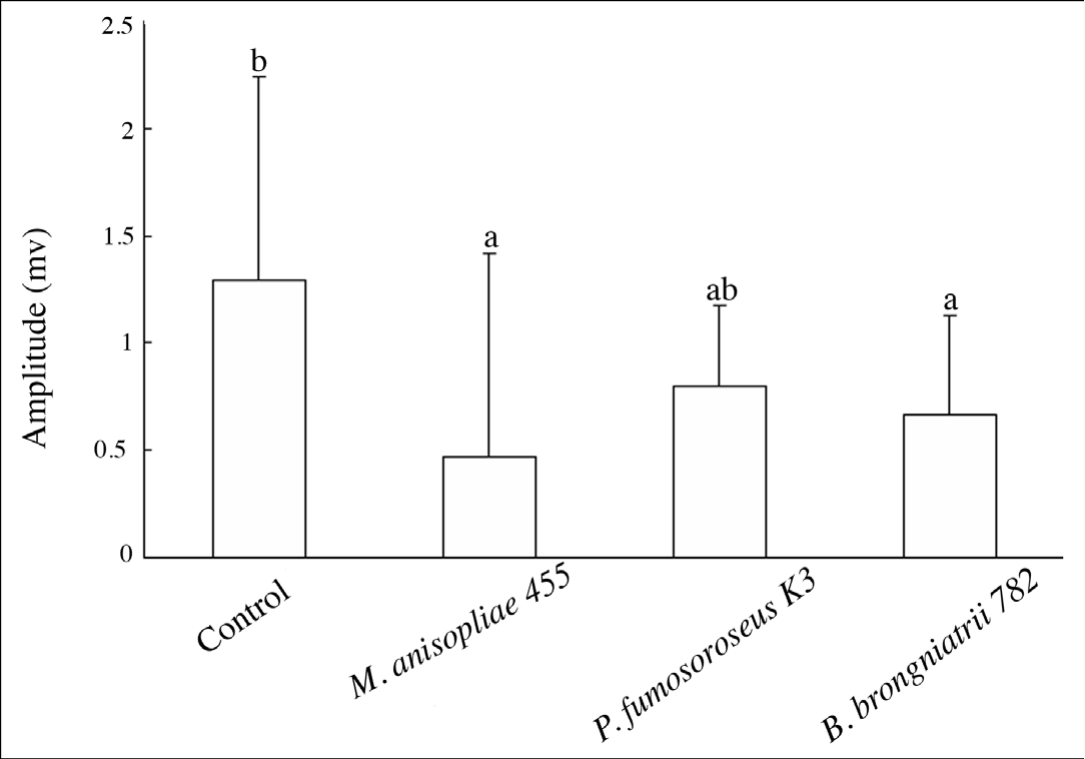
EAG responses of *C. formosanus* to odor originating from suspensions of three species of entomopathogenic fungi and Tween 20 (control). The ertical bars represents standard errors. The letters on the bar give the significance according to the Tukey-Kramer HSD test (n = 25 for each group, p < 0.05).

The average EAG amplitudes of each solution is shown in Figure 3. The EAGs to entomopathogenic fungi were significantly different in magnitude from the control response. The relation between conidia concentration and the EAG response magnitude was examined for *M. anisopliae* 455. The magnitude of the EAG response was taken as the value 1 second after stimulus onset. The response magnitude was almost proportional to the logarithmic value of conidia concentration in the range from 10^3^ to 10^7^ /ml. The response magnitudes fell into 3 significantly different classes between 10^3^ and 10^7^ in the TukeyKramer HSD test (p < 0.05) as shown in Table 6.

### Preference tests

In food preference tests a filter paper discs were used containing 50 µ“1 of 1.0 × 10^7^ conidia/ml fungal suspension to determine if the termites showed a preference for any of the three species of fungi using the method of Ohmura et al. (2006). Food preference was measured by consumption rate (%) of the filter paper disc containing 0.025% Tween 20 solution (control) versus conidial suspensions. The results showed that the termites consumed more *P. fumosoroseus* K3 (66.9 %), than the other two fungi, *B. brongniartii* 782 (47.3%) and *M. anisopliae* 455 (13.9%). These results show that the termites can discriminate between these suspensions at the behavioral level and have distinctly different responses to them (Table 7).

## Discussion

The benefit of mutual grooming behavior against disease susceptibility has been reported in some species of termites (Boucias et al. 1996; Rosengaus and Traniello 2001; Shimizu and Yamaji 2003; Yanagawa and Shimizu 2005). However, the cues stimulating initiation and continuation of grooming behavior remain unclear in many eusocial insects including termites. Though it is well known that many kinds of social behaviors occur after antennal contacts with one another, few papers have dealt with the role of antennae in relation to grooming behavior in termites. Kramm and West (1982) indicated that termites exposed to *M. anisopliae* groomed more extensively than unexposed termites did, suggesting that mutual grooming behavior was initiated by the detection of foreign organisms on their body surface. The termites remove conidia attached to their body surface mainly by mutual grooming (Yanagawa and Shimizu 2005). The termites without antennae all died much faster due to the infection of *M. anisopliae* than the termite with antennae did (Table 5). Furthermore, the termites without antennae were different from the intact termites in details of conidia removal as the site from which the conidia were preferentially removed, the length of time during which conidia removing lasted and the variation of removal rate. Taking all these facts into consideration, termites remove the conidia from the body surface by mutual grooming that appears to protect them from conidial infection, and the antennae may play important roles in the mutual grooming behavior. Namely, the antennae are utilized for selecting grooming sites of nestmates, for controlling the grooming occurrence and for persistence in grooming.

The antennae do not appear to be necessarily essential for the initiation of mutual grooming behavior because mutual grooming occurred not only in termites with antennae but also in those without antennae. On the other hand, the termite eats foreign substances on the body surface using glossae that are part of the labium, on which various types of sensilla are present (Yanagawa and Shimizu 2007). Though these sensilla have not been examined physiologically in termites, some of the sensilla are likely to be chemosensory, because many species of insects have chemosensory sensilla on the mouth parts (Steinbrecht 1999). Thus, the sensory information necessary for initiation of the grooming may come not only from antennal sensory organs but also from sensory organs on other appendages such as labium.

The EAG is a kind of summated potential that includes receptor potentials and action potential of many antennal olfactory receptors excited by odor stimuli. As the odor stimuli used in this study originated from conidial suspensions, they are mixtures of various volatile substances. The kinds, concentration and combination of these volatile substances must be specific to the species of fungi. In our experiments, the termite antennae responded to the odor originating from the fungi with EAGs that were specific to the species of fungi used (Figure 2). These results show that antennal olfactory receptors can detect the odors of these fungi, and suggest that they may have ability to discriminate the species of the fungi by their species-specific odors at leasts under experimental conditions.

As for the behavioral responses to fungi, termites removed conidia of *P. fumosowseus* K3 and *B. bwngniartii* 782 more rapidly than those of *M. anisopliae* 455 (Yanagawa et al. 2007). Exposing termites to filter paper with fungi showed that the termites were capable of distinguishing the three fungi. The EAG responses increased with increasing concentrations of the suspension in the range from 10^3^ to 10^7^ conidia/ml (Table 6). The density of *M. anisopliae* in the soil range from 3.2 × 10^3^ to 7.6 × 10^5^/g dried soil in Japan (Yaginuma, 1990). Thus, the antennal olfactory receptors of the termite work effectively within the normal range of conidial concentration in soil. In addition, as the LD50 of *M. anisopliae* 455 was 6.8 × 10^3^ CFUs/ml when the termites were reared individually (Yanagawa and Shimizu 2005), the induction of grooming behavior might not always depend on the response magnitude of antennal olfactory receptors provided that termites detect the odor of fungi. Further study is needed in order to fully clarify the initiation and continuation of the grooming behavior of termites.

**Table 6.**
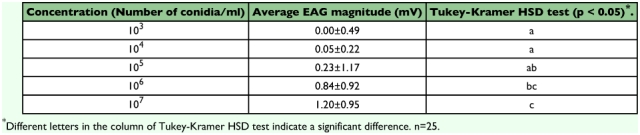
Dose-reacted EAG response magnitude of M. anisopliae 455 odor

**Table 7.**
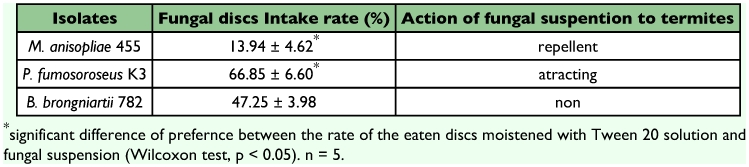
Feeding Preference Tests

## Abbreviations

EAG: electroantennogram
FITC: fluorescent isothiocyanate
